# Polyacetylenes and sesquiterpenes in Chinese traditional herb *Atractylodes lancea*: biomarkers and synergistic effects in red secretory cavities

**DOI:** 10.1186/s43897-024-00130-2

**Published:** 2025-02-04

**Authors:** Daiquan Jiang, Huaibin Lin, Zhenhua Liu, Keke Qi, Wenjin Zhang, Hongyang Wang, Chengcai Zhang, Lu Zhu, Jiaojiao Zhu, Yan Zhang, Luqi Huang, Sheng Wang, Yang Pan, Lanping Guo

**Affiliations:** 1 State Key Laboratory for Quality Ensurance and Sustainable Use of Dao-di Herbs, National Resource Center for Chinese Materia Medica, China, Academy of Chinese Medical Sciences , Beijing, 100700 PR China; 2 Joint Center for Single Cell Biology, Shanghai Collaborative Innovation Center of Agri-Seeds, School of Agriculture and Biology, Shanghai Jiao Tong University, Shanghai, 200240 China; 3 National Synchrotron Radiation Laboratory, University of Science and Technology of China, Hefei, 230029 China; 4 College of Pharmacy, Ningxia Medical University, Yinchuan, 750004 China; 5 Key Laboratory of Biology and Cultivation of Herb Medicine, Ministry of Agriculture and Rural Affairs, Beijing, 100700 PR China; 6 Agriculture and Biotechnology Center, South China, National Botanical Garden , Chinese Academy of Sciences, Guangzhou, 510645 China

The *Atractylodes lancea* rhizome (AR) stands as a significant source of traditional medicine in East Asia, widely recognized for its therapeutic properties in Traditional Chinese Medicine (TCM) (Chen et al. 2016; Zhang et al. 2021). These include liver protection, blood sugar control, diuretic effects, and anti-hypoxic activities, all attributed to its rich content of sesquiterpenoids (such as hinesol and atractylon) and other medicinal components. These compounds have been shown to have anti-bacterial, anti-tumour, and blood sugar-lowering effects, with some, such as hinesol, demonstrating promise against lung cancer (Qin *et al.*, 2019; Acharya et al. 2021a, b; Zhang et al. 2021). The quality of AR varies by region, with the Maoshan variety from Jiangsu, China (JAR), exhibiting the highest medicinal efficacy. The presence of red secretory cavities has long been considered a marker of high quality (Xu et al. 2022; Zhang *et al.*, 2023). However, the specific cause of the colour variation remains unknown.

To investigate the marker metabolites within the distinct-colour secretory cavities (SCs) of AR, we conducted GC-MS analysis to discern the chemical composition variations among the red secretory cavities (RSCs), yellow secretory cavities (YSCs), and non-secretory cavities (NSCs) in JAR (Fig. 1a). Red secretory cavities (RSCs) contained the highest number of metabolites (74), while non-secretory cavities (NSCs) had the lowest (40) (Table S1, Fig S1a, b). Principal component analysis and heatmap clustering confirmed these results. Sesquiterpenes were the most abundant metabolites, with higher levels in RSCs. Further analysis identified unique metabolite compositions among red, yellow, and non-secretory cavities. Nine compounds were shared between red and yellow cavities, potentially contributing to the red coloration (Fig. 1b, S1c-f).

**Fig. 1:**
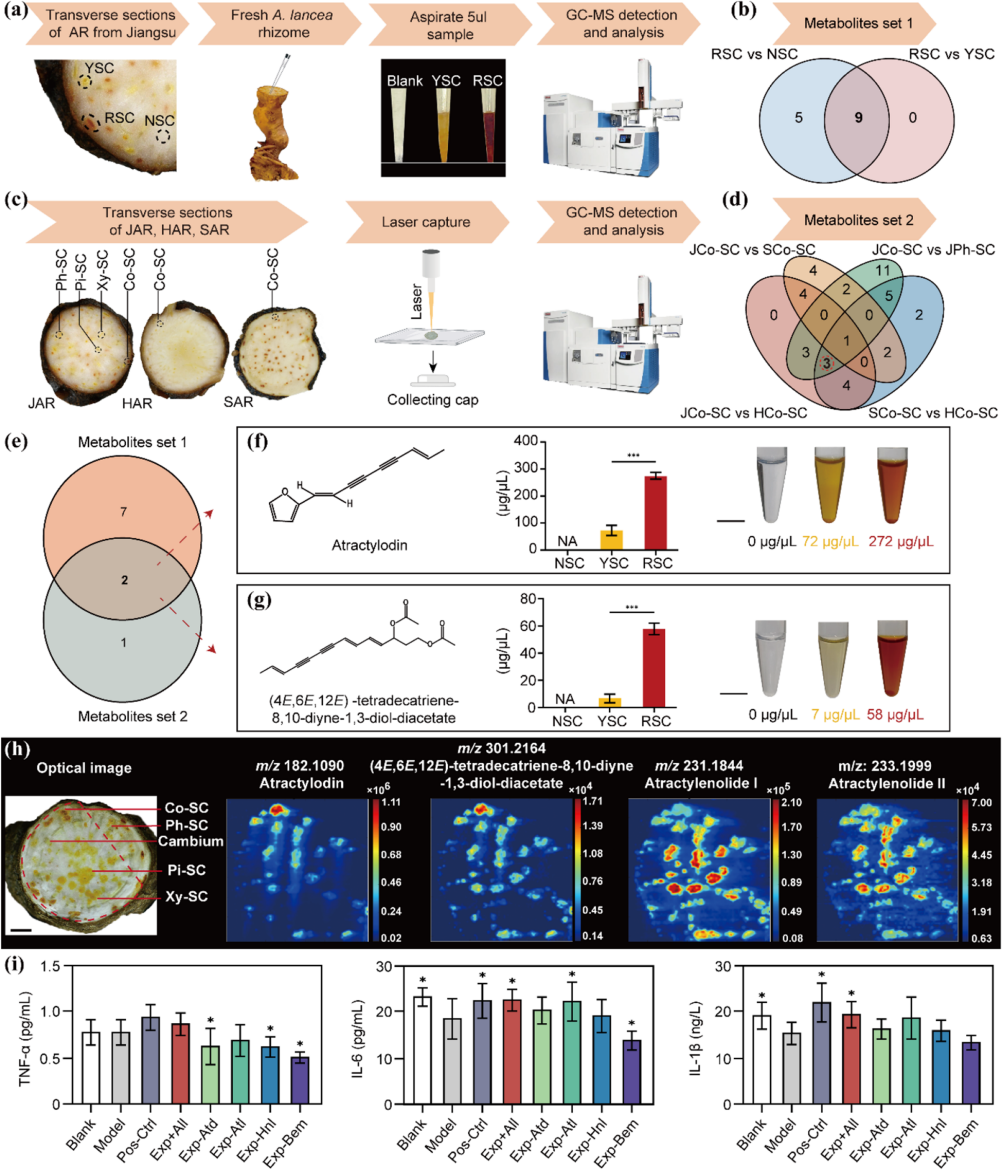
Biomarkers of red secretary cavities and the synergistic effect of polyacetylenes and sesquiterpenes in *Atractylodes lancea*. **a** Overview of sample acquisition and workflow. Red secretory cavities (RSC), yellow secretory cavities (YSC), and non-secretory cavities (NSC) samples were obtained from JAR with three independent biological repeats each. **b** Venn diagram depicting differential metabolites associated with red secretory cavities, highlighting shared and unique compounds among RSC, YSC, and NSC. **c** Overview of sample acquisition and workflow. Samples of AR from three different geographical origins, including transverse sections from Jiangsu (JAR), Shaanxi (SAR), and Hubei (HAR). Secretory cavities in cortex (Co-SC), phloem (Ph-SC), xylem (Xy-SC), and pith (Pi-SC) are indicated. **d** Venn diagram showing the three differential metabolites associated with the red secretory cavities. **e** Venn diagram illustrating the differential metabolites shared between differential metabolite set 1 (9 metabolites presented in Fig. 1b) and set 2 (3 metabolites presented in Fig. 1d). **f**, **g** Structural formulas of atractylodin (**f**) and (4*E*,6*E*,12*E*)-tetradecatriene-8,10-diyne-1,3-diol-diacetate (**g**), and their content differences among RSC, YSC, and NSC (mean ± sem, n=3, *t*-test, ***P < 0.001). Dissolving standard substances were used to simulate the colour differences of atractylodin and (4*E*,6*E*,12*E*)-tetradecatriene-8,10-diyne-1,3-diol-diacetate at different concentrations (concentration reference in quantitative results in (**f**) and (**g**), solvent ethyl acetate). Bars = 1 cm. **h** The optical images of rhizome cross sections of JAR (selected trapezoid patch is marked with red dash line). And corresponding DESI/PI-MSI of selected ions (*m/z*). MS images were recorded with a scanning step size of 200 μm. Bar = 3 mm. **i** Effects of different ratios of essential oil components on the levels of TNF-α, IL-6, and IL-1β in mouse colonic tissue (mean ± sem, n=8). Stars indicate significant differences to the model group (*t*-test, *P* < 0.05). The experimental group Exp+All mirrors the compound composition found in JAR from the geographic region of Geo-herb, featuring 44% atractylon, 37% atractylodin, 8% hinesol, and 11% *ß*-eudesmol. In contrast, Exp-Atd excludes atractylodin, while Exp-Atl omits atractylon. Exp-Hnl eliminates hinesol from the mixture, and Exp-Bem removes ß-eudesmol.

To enable a comprehensive comparison of metabolites across different AR samples, we meticulously selected three representative accessions (specifically JAR, HAR, SAR) exhibiting distinct cavity types from various geographic regions of China (Fig. 1c, S2). To enhance the resolution of chemical distribution in AR, we employed Laser Capture Microdissection (LCM) to isolate the SCs from micro-sections of AR from the three distinct *A. lancea* accessions. The SCs were further categorized by tissue type, encompassing the cortex, phloem, xylem, and pith (Fig. 1c). SCs contained more chemicals (56) than non-cavity areas (6) (Table S2, Fig. S3). SCs from accessions in different regions (HAR, JAR, SAR) had unique metabolic fingerprints. Orthogonal Partial Least Squares Discriminant Analysis (OPLS-DA) was used to identify metabolites that differed between red and non-red SCs (Fig. S4). The analysis showed a clear separation between the different comparison groups, suggesting unique metabolites in red SCs. Three specific polyacetylenes were consistently found in red compared to non-red SCs, and not in comparisons between different types of red SCs (Fig. 1d). This suggests that these three compounds are strongly associated with the red colour of the SCs. Further targeted analysis confirmed the presence of two of these polyacetylenes (atractylodin and (4*E*,6*E*,12*E*)-tetradecatriene-8,10-diyne-1,3-diol-diacetate) in red SCs (Fig. S5).

Utilizing the two identified datasets of differential metabolites from the secretory and non-secretory compartments of AR (Fig. 1b, d), we conducted a quantitative analysis on two common polyacetylenes (Fig. 1e). These included atractylodin (272.72 μg/μL and 72.34 μg/μL in YSC and RSC, respectively) and (4*E*,6*E*,12*E*)-tetradecatriene-8,10-diyne-1,3-diol-diacetate (58 μg/μL and 7 μg/μL in YSC and RSC, respectively). By simulating the actual concentrations within the secretory cavities using corresponding standard substances, the experiment confirmed that both metabolites are responsible for the red coloration in the SCs (Fig. 1f, g). The accumulation of differences in their concentrations directly caused the observed colour differences (red and yellow). This illustrates that the variation in the surface colour of AR is primarily attributed to differences in polyacetylene content. Furthermore, we collected SCs after LCM, extracted metabolites with 80% methanol, and analysed by LC-MS which is commonly used for analysing polar compounds. In total, 43 compounds were detected. Multivariate data analysis revealed no correlation between polar metabolites and red SCs (Table S3, Fig. S6). Altogether, our data indicated that the three non-polar polyacetylenes are the likely causal compounds related to the red SCs in *A. lancea* natural accessions.

We subsequently investigated whether the two polyacetylenes, atractylodin and (4*E*,6*E*,12*E*)-tetradecatriene-8,10-diyne-1,3-diol-diacetate, exhibited high *in situ* accumulation within the red SCs. Focusing on samples from JAR, which harbours two distinct SC types, particularly the cortex SCs with red secretory cavities, we used DESI/PI-MSI for spatial visualization of the major differential metabolites in frozen sections of JAR. Both atractylodin (*m/z* 183.0807) and (4*E*,6*E*,12*E*)-tetradecatriene-8,10-diyne-1,3-diol-diacetate (*m/z* 301.2164) revealed a strong correlation with the coloured SCs in JAR (Fig. 1h). The DESP/PI-MSI result for other polyacetylenes and sesquiterpenes are presented in Fig. S7. In conclusion, our results demonstrate that the high accumulation of atractylodin and (4*E*,6*E*,12*E*)-tetradecatriene-8,10-diyne-1,3-diol-diacetate is likely responsible for the cinnabar-coloured spots in AR.

To date, several bioactive metabolites have been identified in AR, including atractylodin, atractylon, hinesol, and *β*-eudesmol (Zhang et al. 2021; Chen *et al*. 2023). In this study, we specifically identified atractylodin as a biomarker for the red secretory cavities. This raises the question of whether the primary medicinal effect of AR is mainly attributed to atractylodin itself or to a synergistic interaction between atractylodin and other compounds. To explore the impact of the key active components of *A. lancea* on digestive disorders, we utilized an ICR mouse model and treated them with different active compounds from AR in specific proportions (Table S4).

After a successful modelling period, mice in the experimental groups with different ratios of *A. lancea* essential oil demonstrated improvements in mental status, glossy fur, increased food intake, and enhanced activity, along with well-formed faeces (Fig. S8a, b). The body weight of mice in all groups gradually increased, with the most notable effects observed in experimental groups Exp+All and Exp-Atd. Additionally, the faecal moisture content of mice in each group exhibited a gradual increase, with a significant improvement in groups Exp+All and Exp-Bem compared to the modelling group (Fig. S8c).

Following the final administration, we assessed the serum levels of MTL, GAS, and VIP (Fig. S8d-f). Additionally, we measured three pro-inflammatory cytokines commonly associated with spleen deficiency: IL-1β, IL-6, and TNF-α (Fig. 1i). Compared to the blank control group, the model group exhibited significantly increased serum levels of MTL, GAS, and VIP, accompanied by decreased levels of IL-6 and IL-1β. Compared to the model group, GAS levels were restored in groups Exp-Atd, Exp-Atl, and Exp-Bem, while VIP levels were restored in most treatment groups except for group Exp-Atl. Notably, the treatment resembling JAR from the geographic region of Geo-herb (group Exp+All) significantly rescued both IL-6 and IL-1β. Furthermore, we assessed the pathological changes in the colon using Haematoxylin and Eosin (HE) staining. Mice in experimental group Exp+All showed the most noticeable improvement in the arrangement and structural integrity of glandular and goblet cells within the colonic lumen (Fig. S8g). Collectively, our findings indicate that the optimal ratio of bioactive compounds, resembling that found in Geo-herbs, determines the best medicinal effect.

In summary, we systematically analysed two representative *A. lancea* natural accessions, each characterized by distinct secretory cavities. The results revealed two polyacetylenes, atractylodin and (4*E*,6*E*,12*E*)-tetradecatriene-8,10-diyne-1,3-diol-diacetate, that were significantly associated with the distinctive red coloration of secretory cavities in *A. lancea*. Although we employed various metabolomic methods, we acknowledge that there may be other important metabolites responsible for the coloration and medicinal efficacy due to the limitations of these methods and the complexity of TCM samples. Using a mouse model of diarrhoea induced by spleen deficiency, we further investigated whether the medicinal efficacy of AR is primarily attributed to atractylodin alone or to a synergistic interaction with other compounds. The results indicated that the authentic chemical composition of *A. lancea* significantly ameliorates symptoms of spleen deficiency-induced diarrhoea in mice, with atractylodin playing a substantial role in the therapeutic effect. Our findings provide unprecedented insights into the molecular basis underlying the most optimal phenotype (cinnabar-like red secretory cavities) and the Geo-herbalism in TCM.

## Supplementary Information

Supplementary Material 1

Supplementary Material 2.

Supplementary Material 3

## Data Availability

All datasets generated for this study are included in the manuscript and/or the Supplementary Files.
