# Supplementary material for: Polyacetylenes and sesquiterpenes in Chinese traditional herb Atractylodes lancea: biomarkers and synergistic effects in red secretory cavities
Source: Mol Hortic. 2025 Feb 4;5:11. doi: 10.1186/s43897-024-00130-2 (PMC11792185; doi:10.1186/s43897-024-00130-2)
Supplement: Supplementary file 2 — Supplementary Material 2. [file 43897_2024_130_MOESM2_ESM.docx]

**Polyacetylenes and Sesquiterpenes in Chinese traditional herb *Atractylodes lancea*: Biomarkers and Synergistic Effects in Red Secretory Cavities**

**Daiquan Jiang^a,e,f#^, Huaibin Lin^a#^, Zhenhua Liu^b#^, Keke Qi^c#^, Wenjin Zhang^d^, Hongyang Wang^a,e^, Chengcai Zhang^a,e^, Lu Zhu^b^, Jiaojiao Zhu^b^, Yan Zhang^a,e^, Luqi Huang^a,e^, Sheng Wang^a,e*^, Yang Pan^c*^, Lanping Guo^a,e*^**

^a^State Key Laboratory for Quality Ensurance and Sustainable Use of Dao-di Herbs, National Resource Center for Chinese Materia Medica, China Academy of Chinese Medical Sciences, Beijing, 100700, PR China

^b^Joint Center for Single Cell Biology; Shanghai Collaborative Innovation Center of Agri-Seeds, School of Agriculture and Biology, Shanghai Jiao Tong University, Shanghai 200240, China

^c^National Synchrotron Radiation Laboratory, University of Science and Technology of China, Hefei 230029, China

^d^College of Pharmacy, Ningxia Medical University, Yinchuan 750004, China

^e^Key Laboratory of Biology and Cultivation of Herb Medicine, Ministry of Agriculture and Rural Affairs, Beijing, 100700, PR China

^f^Agriculture and Biotechnology Center, South China National Botanical Garden, Chinese Academy of Sciences, Guangzhou, 510645, China

* Corresponding authors. Tel: +86 10 64087921; fax: +86 10 64081600

E-mail addresses: [mmcniu@163.com](mailto:mmcniu@163.com) (Sheng Wang), [panyang@ustc.edu.cn](mailto:panyang@ustc.edu.cn) (Yang Pan), [glp01@126.com](mailto:glp01@126.com) (Lanping Guo).

# Daiquan Jiang, Huaibin Lin, Zhenhua Liu, and Keke Qi contribute equally to this work.

**Running title:** **Polyacetylenes: Indicators of Red Secretory Cavities in *Atractylodes lancea* Rhizome**

# Materials and methods

*Plant materials and reagents*

All *Atractylodes lancea* accessions were collected from their natural habitats (sources and morphological descriptions are listed in Table S5). All samples were identified by the corresponding author Sheng Wang (Traditional Chinese Medicine Resource Center, China Academy of Chinese Medical Sciences). Chemical standards, including atractylodin, hinesol, *β*-eudesmol, and atractylon (all with a minimum purity of 98%), were purchased from Rongchengxinde (Beijing, China). Additionally, (4*E*,6*E*,12*E*)-tetradecatriene-8,10-diyne-1,3-diol-diacetate was acquired from Sichuan Weikeqi Biological Technology (Sichuan, China), with purity exceeding 98%. All chemicals were of chromatographic grade.

*Sample preparation*

Fresh *Atractylodes lancea* rhizomes (AR) were carefully selected and sectioned open. Essential oil samples, approximately 5 μL each, were extracted from the red secretory cavity (RSC) and yellow secretory cavity (YSC) using a pipette tip. For the non-secretory cavity (NSC) regions, samples of equal mass were collected for GC-MS analysis. In the case of LCM and DESI/PI-MSI, the fresh AR was shaped into a cylinder (2 cm high) and positioned on plastic embedding rings (Leica, Germany). The rings were fixed with the holding clamp of the rotary microtome (Leica RM2265) and 70 μm thin tissue sections were generated at -18℃. The slices were mounted on polyethylene terephthalate (PET) microscope slides with steel frames (76 × 26 mm, 1.4 µm; Leica Microsystems, Germany) and stored at -20℃. For DESI/PI-MSI, the same approach was employed, but plain glass slides (76 × 26 mm, 1.2 mm; Fan Chuan, China) were used.

*Laser Capture Microdissection*

LCM processing was performed as described previously (Chen *et al.*, 2019; Zhou *et al.*, 2018). The secretory cavity (SC) and non-secretory cavity (NSC) regions in the cortex, phloem, xylem, and pith were dissected using the Laser Capture Microdissection System (Leica Microsystems, Germany). Laser conditions for microdissection were set as follows: aperture set at 37, power of 60 μJ, speed at 11, head current at 100%, and pulse frequency of 3309 Hz. An area of approximately 1 mm^2^ (~6-10 SCs or NSCs) of certain regions was dissected separately for each sample. The microdissected tissues were collected into the caps of 500 µL microcentrifuge tubes (Leica Microsystems) by gravity and stored at -20℃ prior to extraction. We captured 6 replicates for each type of SC and NSC region from different ARs.

*GC-MS analysis*

To extract the essential oil, samples were subjected to ultrasonic extraction with 500 μL of hexane for 30 minutes, followed by centrifugation at 12,000 rpm for 10 minutes. The upper organic phase was collected, filtered through a 0.22 μm membrane, and stored at 4℃ until analysis. Laser-captured samples, sized equivalently to those in LCM, were extracted with 100 μL [hexane](javascript:;). After 60 min of sonication, the sample was centrifuged at 12,000 rpm, 25℃ for 10 min. 90 μL of the supernatant was transferred into a brown vial with a glass insert and stored at 4℃ before analysis. GC-MS analysis was performed on a Thermo-Fisher Scientific gas chromatography (Waltham, MA, USA) combined with a triple quadrupole mass spectrometer system (TP-8030). A DB-5MS (5% phenyl methyl polysiloxane) capillary column (30 m × 0.25 mm, 0.25 μm) with helium as a carrier gas at 1 mL/min was employed. The column temperature was programmed initially at 120℃, and then increased to 200℃ at a rate of 5℃/min, and then increased to 240℃ at a rate of 10℃/min, held for 5 min. The mass spectrometer operated in electron ionization (EI) mode at 70 eV, with an interface temperature and ion source temperature at 250℃. Scanning occurred continuously from 50 to 550 atomic mass units (amu). Peaks were identified by comparing the mass spectra data with the National Institute of Standards and Technology (NIST) spectral library. Standards were accurately weighed and dissolved in hexane at a concentration of 0.5 mg/mL. Various concentrations were derived from each standard solution by dilution with hexane to establish calibration curves (Table S6). The limit of detection (LOD) and limit of quantitation (LOQ) were determined by injecting a series of diluted standard solutions until the signal-to-noise (S/N) ratio was 3 for LOD and 10 for LOQ in all quantitative analyses.

*LC-MS analysis*

For LC-MS analysis sample preparation, the same methodology as described for GC-MS was applied, with the exception that extraction was performed using 100 μL of [80%](javascript:;) v/v methanol. LC analysis was performed on a Waters Acquity UPLC-I-Class system (Waters Corp., Milford, MA, US) with an Acquity HSS T3 column (100 × 2.1 mm, 1.8 μm) for chromatographic separation (Shan *et al.*, 2014). The column temperature was set at 25℃, the flow rate at 0.3 mL/min, and the injection volume was 5 μL for each sample. The mobile phase consisted of 0.1% formic acid aqueous solution (A) and acetonitrile containing 0.1% formic acid (B). The gradient elution program was as follows: 0~3 min, 2%~8% B; 3~5 min, 8%~15% B; 5~11 min, 15%~35% B; 11~13 min, 35%~65% B; 13~15.5 min, 65%~100% B; 15.5~16 min 100%~2% B. After UPLC, the effluent was alternatively connected to a Xevo G2-SQ-TOF MS system (Waters Corp., Milford, MA, US). The data acquisition mode was MS^E^ continuum. The experiment was performed in both the ESI (+) and ESI (–) ionization modes. The parameters were set as follows: source and desolvation temperatures at 100℃ and 350℃ respectively; desolvation gas flow rate at 900 L/h; capillary voltage at 0.5 kV for ESI (+) and 2 KV for ESI (–); cone voltage at 40 V; collision energy at 6 eV (trap) for low-energy scans, 20-30 eV and 45-70 eV for ESI (–) and ESI (+) in high-energy scans respectively. The data acquisition range spanned from 50 to1500 Da. The mass accuracy was maintained using a lock spray with leucine enkephalin (200 pg/μL, 10 μL/min) as the reference.

*DESI/PI-MSI*

The experimental procedure for DESI/PI-MSI closely followed the methodologies detailed in previous studies (Liu *et al.*, 2021; Liu *et al.*, 2019). Samples were prepared as described before. The metabolite distribution was conducted using a modified Agilent 6224 Accurate-Mass TOF Mass Spectrometer (Agilent, USA). Data storage synchronization occurred with the activation of stepper motor for every line scanned, facilitated by custom-developed software based on LabVIEW (Bitter *et al.*, 2006). For imaging, using the open source ProteoWizard converters, the collected Agilent Chemstation.D files were converted to “.mzML” format files, which were then merged into one “.imzML” format file for image reconstruction with imzMLConverter (Race *et al.*, 2012). All ion image reconstructions, background subtraction and the generation of an average mass spectrum in a region of the sections were performed using the freely available standalone version of the MSiReader software. Key parameters included X and Y pixel sizes at 200 μm, a raster speed of 370 μm/s, a spray solvent composed of 70% MeOH, 30% Toluene, 1% Formic acid, with flow rate at 3 μL/min. MS settings involved positive polarity, a 3.5 kV capillary voltage, 80 V cone voltage, 1.2 MPa pressure of nitrogen as carrier gas, and mass range of *m/z* 20-1000. Imaging mass spectra of available standards (atractylodin & atractylon) are shown in Fig. S9.

*Animal housing and drug preparation*

Eighty male SPF-grade ICR mice, with a body weight of 18-22 grams, were housed at the Pharmacology Platform of Ningxia Medical University (protocol number: SCXK (Ning) 2020-0001). The mice were maintained under controlled conditions with a temperature of 20-25°C and relative humidity of 40%-60%, subjected to a 12-hour light-dark cycle. Free access to food and water was provided, with regular feed and bedding materials provided by the Experimental Animal Center of Ningxia Medical University. For the rhubarb extract preparation, 100 grams of raw rhubarb (Ningxia Golden Sun Pharmaceutical Co., LTD., lot number: 20230301) were soaked in 500 mL of water (5 times the weight of rhubarb) for 30 minutes. Subsequently, it was boiled at 90°C for 1 hour and concentrated to 1 g/mL. The extract was stored at 4°C for later use. To determine the mouse dosage equivalent to the human dose of Shenlingbaizhu granules (Yunnan Tengyao Pharmaceutical Co., LTD., lot number: 20230544), which is administered at 6 g per time, three times a day, a conversion was made, resulting in a dosage of 2249 mg/kg for mice. The Shenlingbaizhu solution was then prepared, considering the gavage dose of 0.1 mL per 10 g of body weight.

*Grouping, modelling and drug treatment of animals.*

After one week of adaptation feeding, mice were randomly divided into 8 groups, each consisting of 10 mice. The groupings were as follows: the blank control group, model group, positive control group, and experimental groups (Exp+All, Exp-Atd, Exp-Atl, Exp-Hnl, Exp-Bem) (see Table S4 for the ratio of essential oil components in each group). Throughout a consecutive 7-day period, all groups, except for the blank control group, were subjected to gavage administration of 0.2 mL/10 g Rheum palmatum extract twice daily. The blank control group received 0.1 mL/10 g of physiological saline via gavage. The positive control group received 0.1 mL/10 g of Shenlingbaizhu solution by gavage, which is a typical Chinese patent medicine for drying dampness and strengthening the spleen. The experimental groups were gavage-administered a mixture according to the ratio and dosage of essential oil components at 100 mg/kg for the same 7-day duration.

*Body composition analysis and stool detection*

The body weight of the mice was recorded on the 1st, 3rd, 5th, and 7th days during both the modelling and administration periods. During this time, observations were made on changes in body condition, behaviour, activity, food intake, and perianal area of the mice in each group. On the 1st, 3rd, 5th, and 7th days of modelling, and on the last day of administration, each group of mice was placed in metabolic cages after oral gavage. Mouse feces were collected within a 5-hour period. The wet weight of feces (W1) from each group within the 5-hour period was recorded. The collected feces were then placed in a culture dish and dried in a thermostatic drying oven at 105°C for 20 minutes to obtain the dry weight (W2). The fecal moisture content was calculated as [(W1-W2)/W1] × 100%.

*Gastrointestinal motility test, inflammation level detection and histopathological examination of colon*

After the final administration, the mice were fasted but allowed access to water for 24 hours. Blood was collected in centrifuge tubes and allowed to stand for 1-2 hours. After centrifugation at 4°C and 3000 rpm for 10 minutes, the supernatant was separated and stored at -20°C for future use. The levels of Gastrin (GAS), Vasoactive Intestinal Peptide (VIP), and Motilin (MTL) in the serum were determined using ELISA kits (Jiangsu Jingmei Biotechnology Co., LTD, lot number: 202308), following the instructions provided in the kit. After completion of the administration, the proximal colon tissue of the mice was collected, minced, weighed, and mixed with physiological saline (tissue: saline = 1 g: 9 mL). The mixture was homogenized using a homogenizer immersed in ice water for 6-8 minutes. After centrifugation at 4°C and 3000 rpm for 15 minutes, the supernatant was collected. The levels of IL-6, IL-1β, and TNF-α were measured following the instructions provided in the reagent kit. Approximately 1 cm of the proximal colon tissue from the cecum was washed with physiological saline, fixed in 4% paraformaldehyde for 24 hours, embedded in paraffin using standard procedures, sectioned, and stained with haematoxylin and eosin (HE). The histopathological changes in the colon tissue of the mice from each group were observed under a microscope.

*Statistical analysis*

The Xcalibur software (Thermofisher Co., Milford, MA, USA) was used for mass spectrum analysis (Kang *et al.*, 2020). Due to the trace quantity and potential interference from the tissue embedding agent and PET membrane, a targeted extraction of characteristic ions was employed to quantify the trace compounds in Laser Capture Microdissection (LCM) samples. Initially, 56 compounds from the three geographic origins were identified through direct extraction. Subsequently, each compound in the LCM samples underwent quantitative analysis through the extraction of a single characteristic ion.

Relative quantification of identified compounds in samples for comparison was based on the mean ionic intensity of the characteristic ions (Lovestead and Urness *et al*., 2020). The hierarchical cluster analysis (HCA) results were demonstrated as heatmaps with dendrograms. The Pearson correlation coefficients (PCC) were calculated using the clustering function. Heatmaps were generated using the pheatmap package in R. Principal component analysis (PCA) and orthogonal partial least squared discriminant analysis (OPLS-DA) were conducted to evaluate the differences in chemical profiles of groups using the EZinfo software 3.0 (Version 3.0; Waters Co., Milford, MA). Logarithmic transformation was applied to all data, following the methodology outlined previously (Lyu *et al.*, 2020). All statistical analyses were performed using SPSS Statistics 20.0. Statistically significant differences were determined by Student's *t-*test, and *P* values less than 0.05 were considered statistically significant in all cases.

# References

**Bitter, R.M., T.; Nawrocki, M.** 2006. LabVIEW: Advanced programming techniques (Crc Press). <https://doi.org/10.1201/9780849333255>.

**Chen, L.L., Chu, S.S., Zhang, L., Xie, J., Dai, M., Wu, X., and Peng, H.S.** Tissue-Specific Metabolite Profiling on the Different Parts of Bolting and Unbolting Peucedanum praeruptorum Dunn (Qianhu) by Laser Microdissection Combined with UPLC-Q/TOF(-)MS and HPLC(-)DAD. Molecules. 2019;24:1439. <https://doi.org/10.3390/molecules24071439>.

**Kang, C., Lv, C., Yang, J., Kang, L., Ma, W., Zhang, W., Wang, S., Wang, T., Sun, J., Ge, Y., et al.** A Practical Protocol for a Comprehensive Evaluation of Sulfur Fumigation of Trichosanthis Radix Based on Both Non-Targeted and Widely Targeted Metabolomics. Front Plant Sci. 2020;11:578086. <https://doi.org/10.3389/fpls.2020.578086>.

**Liu, C., Qi, F., and Pan, Y.** Imaging of Polar and Nonpolar Lipids Using Desorption Electrospray Ionization/Post-photoionization Mass Spectrometry. Methods in molecular biology. 2021;2306:285-298. https://doi.org/10.1007/978-1-0716-1410-5_19.

**Liu, C., Qi, K., Yao, L., Xiong, Y., Zhang, X., Zang, J., Tian, C., Xu, M., Yang, J., Lin, Z., et al.** Imaging of Polar and Nonpolar Species Using Compact Desorption Electrospray Ionization/Postphotoionization Mass Spectrometry. Analytical Chemistry. 2019;91:6616-6623. <https://doi.org/10.1021/acs.analchem.9b00520>.

**Lovestead, T. M., & Urness, K.** Gas Chromatography Mass Spectrometry (GC-MS). National Institute of Standarts and Technology. 2020; <https://tsapps.nist.gov/publication/get_pdf.cfm?pub_id=926655>.

**Lyu, C., Yang, J., Wang, T., Kang, C., Wang, S., Wang, H., Wan, X., Zhou, L., Zhang, W., Huang, L., et al.** A field trials-based authentication study of conventionally and organically grown Chinese yams using light stable isotopes and multi-elemental analysis combined with machine learning algorithms. Food Chem. 2020;343:128506. <https://doi.org/10.1016/j.foodchem.2020.128506>.

**Race, A.M., Styles, I.B., and Bunch, J.** Inclusive sharing of mass spectrometry imaging data requires a converter for all. Journal of Proteomics. 2012;75:5111-5112. <https://doi.org/10.1016/j.jprot.2012.05.035>.

**Shan, G.S., Zhang, L.X., Zhao, Q.M., Xiao, H.B., Zhuo, R.J., Xu, G., Jiang, H., You, X.M., and Jia, T.Z.** Metabolomic study of raw and processed Atractylodes macrocephala Koidz by LC–MS. Journal of Pharmaceutical and Biomedical Analysis. 2014;98:74-84. <https://doi.org/10.1016/j.jpba.2014.05.010>.

**Zhou, W., Liang, Z., Li, P., Zhao, Z., and Chen, J.** Tissue-specific chemical profiling and quantitative analysis of bioactive components of Cinnamomum cassia by combining laser-microdissection with UPLC-Q/TOF–MS. Chemistry Central Journal. 2018;12:71. https://doi.org/10.1186/s13065-018-0438-x.
