# Supplementary material for: Polyacetylenes and sesquiterpenes in Chinese traditional herb Atractylodes lancea: biomarkers and synergistic effects in red secretory cavities
Source: Mol Hortic. 2025 Feb 4;5:11. doi: 10.1186/s43897-024-00130-2 (PMC11792185; doi:10.1186/s43897-024-00130-2)
Supplement: Supplementary file 3 — Supplementary Material 3 [file 43897_2024_130_MOESM3_ESM.docx]

**Polyacetylenes and Sesquiterpenes in Chinese traditional herb *Atractylodes lancea*: Biomarkers and Synergistic Effects in Red Secretory Cavities**

**Daiquan Jiang^a,e,f#^, Huaibin Lin^a#^, Zhenhua Liu^b#^, Keke Qi^c#^, Wenjin Zhang^d^, Hongyang Wang^a,e^, Chengcai Zhang^a,e^, Lu Zhu^b^, Jiaojiao Zhu^b^, Yan Zhang^a,e^, Luqi Huang^a,e^, Sheng Wang^a,e*^, Yang Pan^c*^, Lanping Guo^a,e*^**

^a^State Key Laboratory for Quality Ensurance and Sustainable Use of Dao-di Herbs, National Resource Center for Chinese Materia Medica, China Academy of Chinese Medical Sciences, Beijing, 100700, PR China

^b^Joint Center for Single Cell Biology; Shanghai Collaborative Innovation Center of Agri-Seeds, School of Agriculture and Biology, Shanghai Jiao Tong University, Shanghai 200240, China

^c^National Synchrotron Radiation Laboratory, University of Science and Technology of China, Hefei 230029, China

^d^College of Pharmacy, Ningxia Medical University, Yinchuan 750004, China

^e^Key Laboratory of Biology and Cultivation of Herb Medicine, Ministry of Agriculture and Rural Affairs, Beijing, 100700, PR China

^f^Agriculture and Biotechnology Center, South China National Botanical Garden, Chinese Academy of Sciences, Guangzhou, 510645, China

* Corresponding authors. Tel: +86 10 64087921; fax: +86 10 64081600

E-mail addresses: [mmcniu@163.com](mailto:mmcniu@163.com) (Sheng Wang), [panyang@ustc.edu.cn](mailto:panyang@ustc.edu.cn) (Yang Pan), [glp01@126.com](mailto:glp01@126.com) (Lanping Guo).

# Daiquan Jiang, Huaibin Lin, Zhenhua Liu, and Keke Qi contribute equally to this work.

**Running title:** **Polyacetylenes: Indicators of Red Secretory Cavities in *Atractylodes lancea* Rhizome**


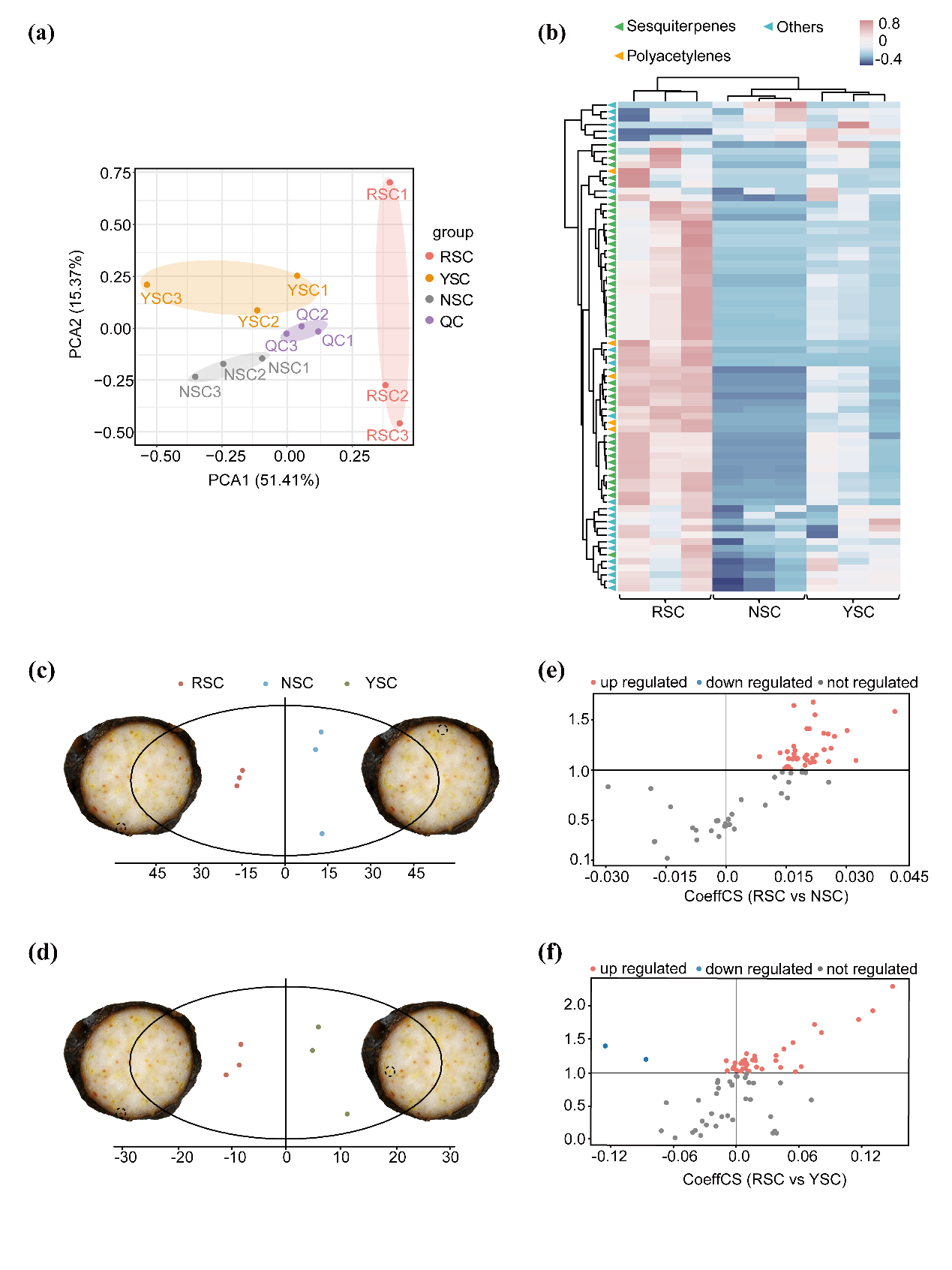


**Figure S1. Metabolomic profiles of secretory cavities (SCs) in natural Jiangsu *Atractylodes lancea* rhizome (JAR).** (a) Principal component analysis (PCA) of SC metabolites determined by GC-MS in three different SC types: red secretory cavities (RSC), yellow secretory cavities (YSC), and non-secretory cavities (NSC). Quality control (QC) represents the mixture of the secretory cavity sample extracts. (b) Clustering heatmap illustrating the relative abundance of metabolites in RSC, YSC, and NSC (n=3). The heatmaps were generated based on min-max normalization of metabolite content. (c, d) OPLS-DA model plots of YSC and NSC compared to RSC (n=3) to screen of key metabolites for red SCs. (e, f) Volcano plots showing the differential metabolites between YSC and NSC compared to RSC. Blue spots show down regulated differentially expressed metabolites, red spots illustrate up regulated differentially expressed metabolites, and black spots represent detected metabolites with insignificant differences.


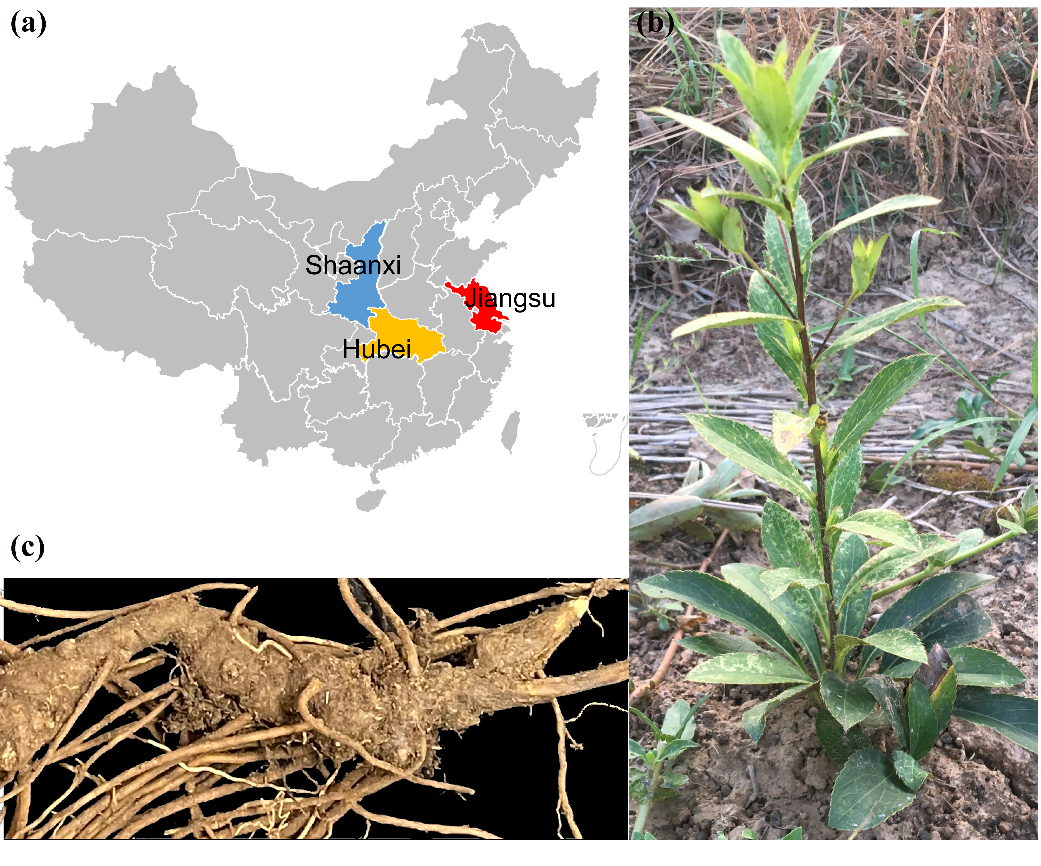


**Figure S2. Geographical origins and appearance of *A. lancea* natural accessions.** (a) Three accessions were collected from different geographic regions in China (Shaanxi, Hubei, and Jiangsu provinces). (b, c) The aboveground (stem and leaf) and underground (root and rhizome) parts of *A. lancea*.


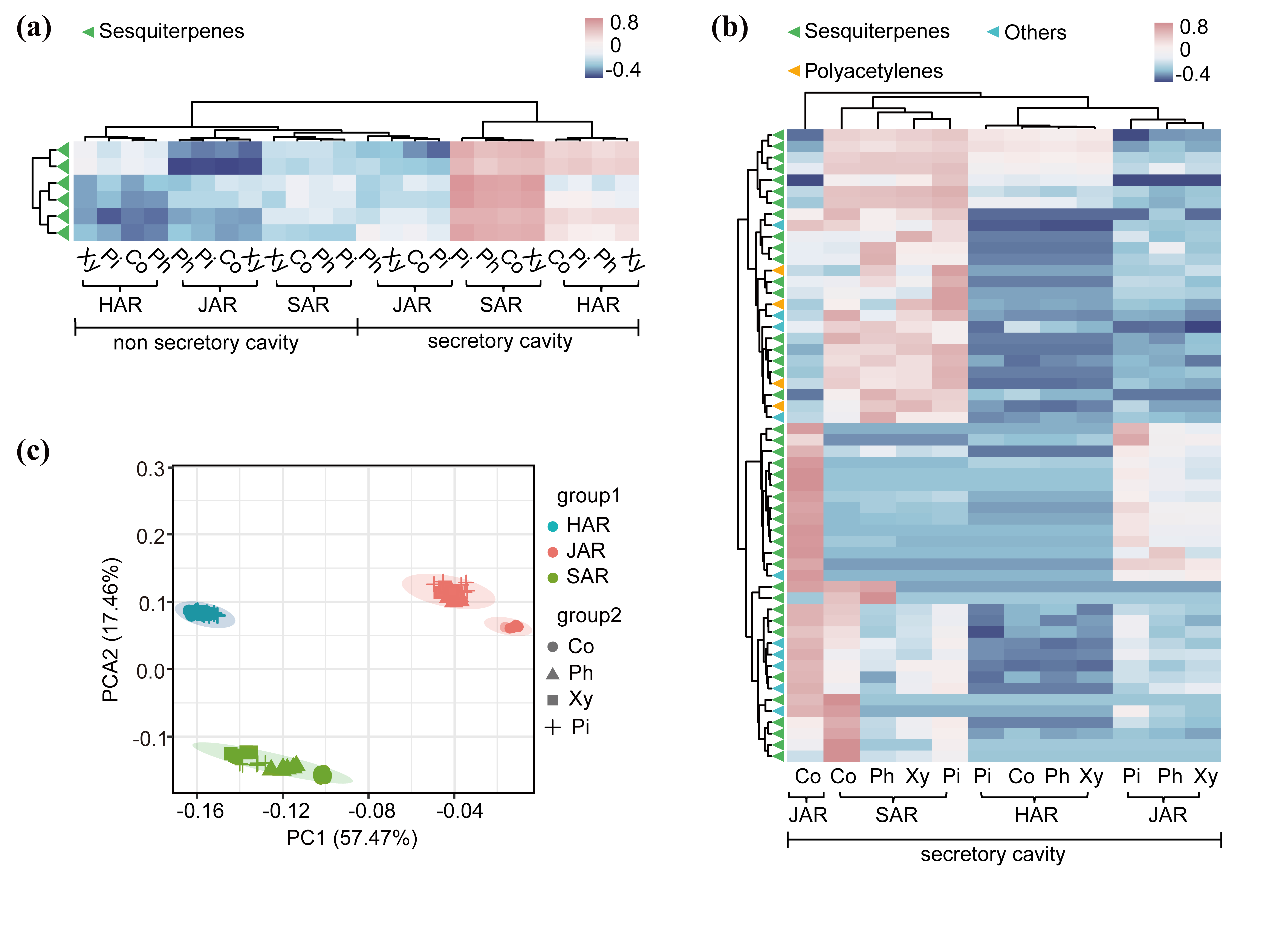


**Figure S3. Comparative metabolomics using Laser Capture Microdissection (LCM) and GC-MS reveals metabolites associated with secretory cavities (SCs) across natural *A. lancea* accessions.** (a) Clustering of metabolites identified in both SCs and non-SCs across three natural accessions. The colour scale indicates the relative change in metabolite levels (n=6). (b) Clustering of SC metabolites identified in different tissues across three natural accessions. The heat maps were generated based on a min-max normalization of metabolite content. The colour scale indicates the relative change in metabolite levels (n=6). (c) Principal component analysis (PCA) of SC metabolites in four different tissues across three natural accessions. Samples of AR from three different geographical origins, including transverse sections from Jiangsu (JAR), Shaanxi (SAR), and Hubei (HAR). Secretory cavities in cortex (Co), phloem (Ph), xylem (Xy), and pith (Pi) are indicated.


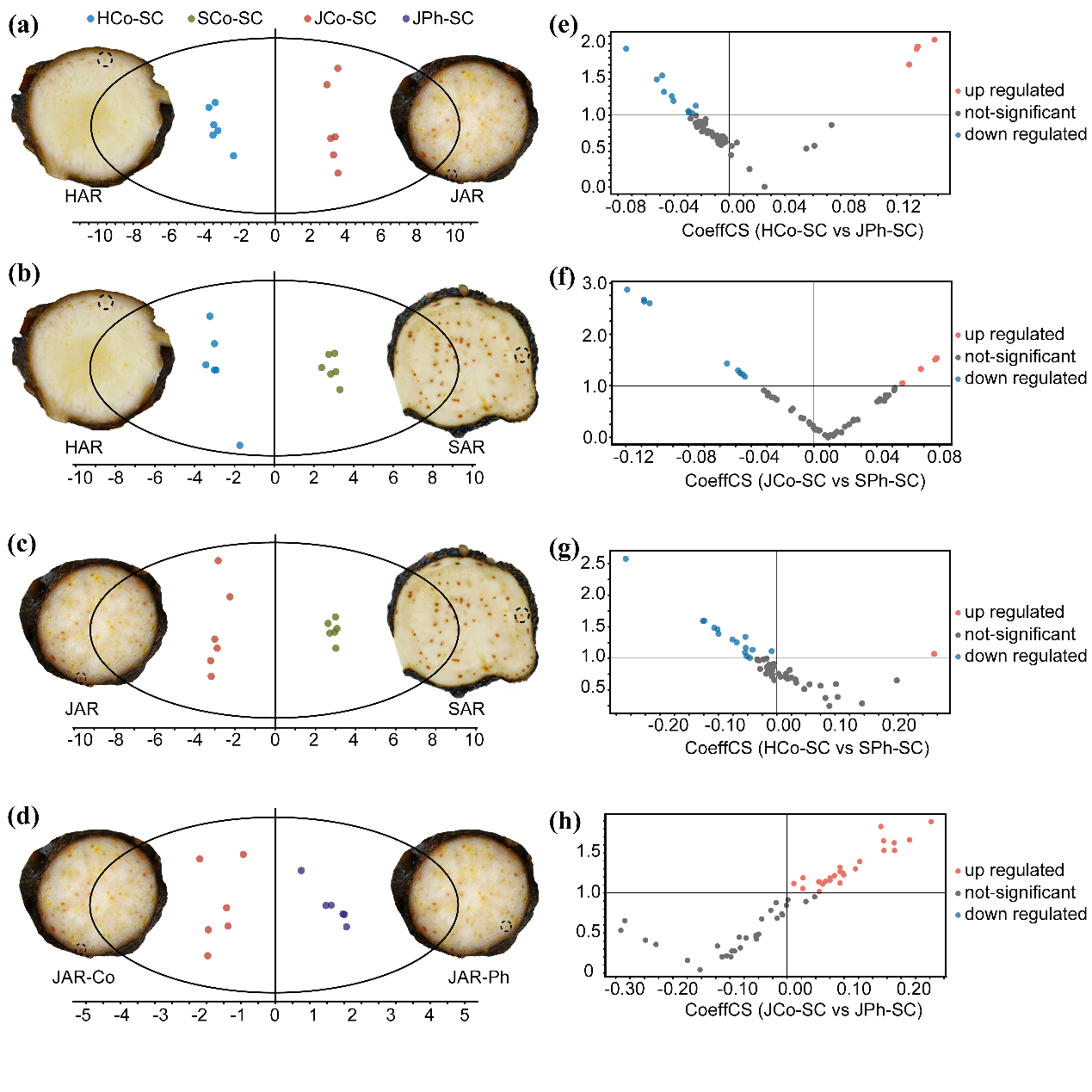


**Figure S4. Screening for biomarkers of red secretory cavities (SCs) in *A. lancea*.** (a-d) Pair-wise comparisons of different SC types using OPLS-DA model plots (n=6). (e-h) Volcano plots showing the differential metabolites in compared samples (VIP ≥ 1). Blue spots show down regulated differentially expressed metabolites, red spots illustrate up regulated differentially expressed metabolites, and black spots represent detected metabolites with insignificant differences. Samples of AR from three different geographical origins, including transverse sections from Jiangsu (JAR, J), Shaanxi (SAR, S), and Hubei (HAR, H). Secretory cavities in cortex (Co-SC), phloem (Ph-SC), xylem (Xy-SC), and pith (Pi-SC) are indicated.


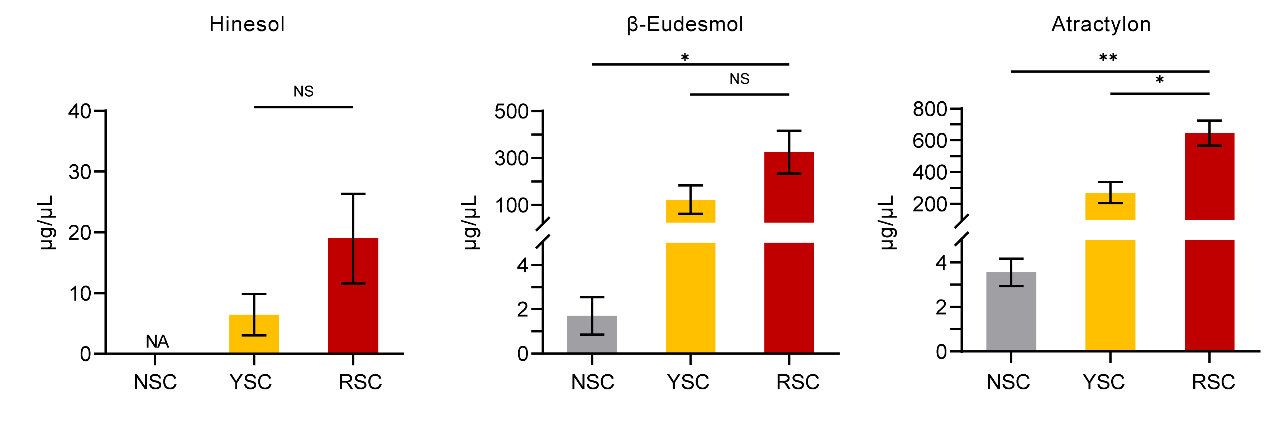


**Figure S5.** Absolute quantitation of 3 representative sesquiterpenes compounds. Hinesol, *ß*-eudesmol, and atractylon levels were determined using GC-MS in red secretory cavities (RSC), yellow secretory cavities (YSC), and non-secretory cavities (NSC) from natural Jiangsu *Atractylodes lancea* rhizome (JAR). Significant differences were determined using a two-tailed Student's *t*-test (mean ± sem, n=3, ^**^*P* < 0.01, ^*^*P* < 0.05). NS, not significant.


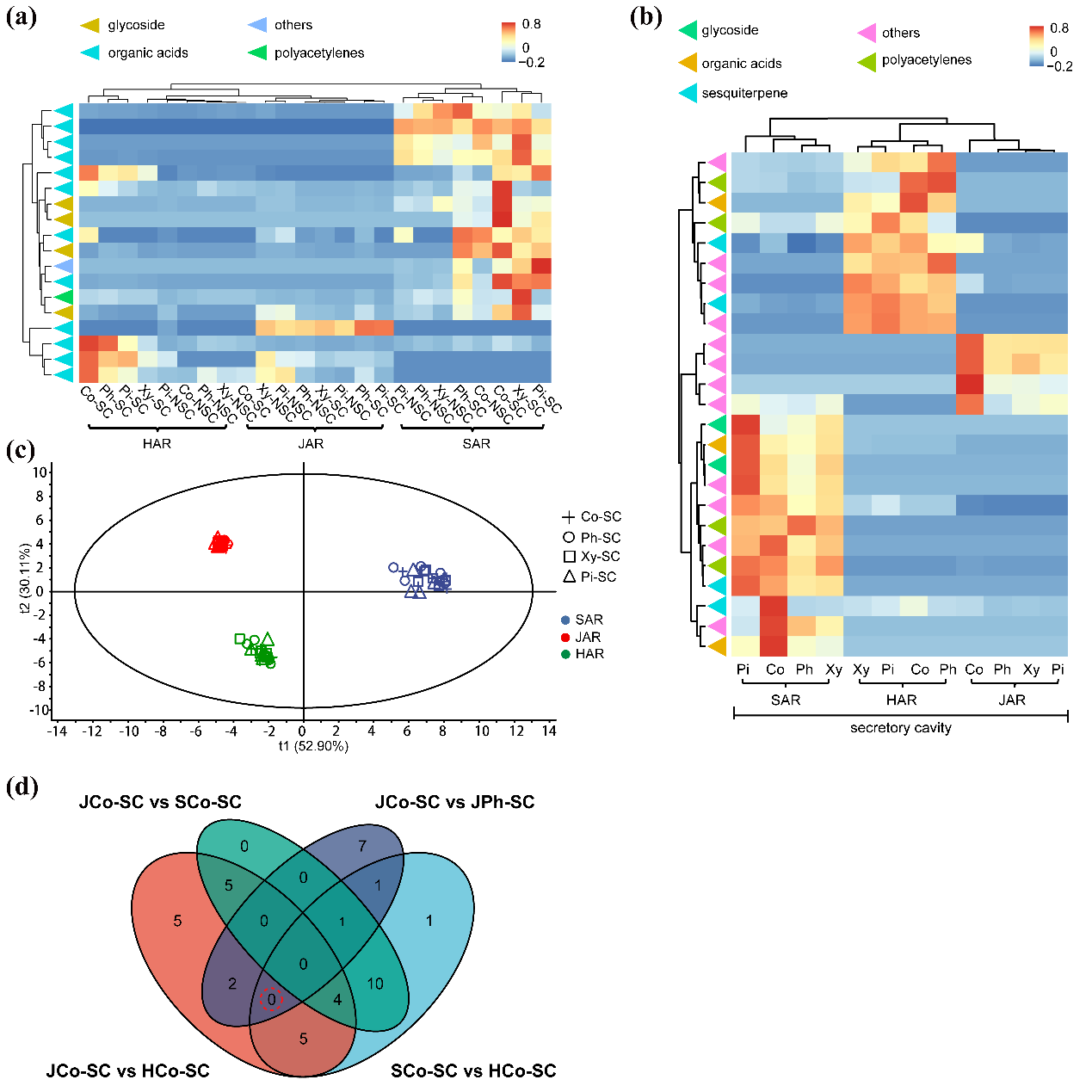


**Figure S6. Comparative metabolomics using Laser Capture Microdissection (LCM) and LC-MS reveals metabolites associated with secretory cavities (SCs) across natural *A. lancea* accessions.** (a) Clustering of metabolites identified in both SC and non-SCs across three natural accessions. The metabolite content was transformed with min-max normalization and visualized by heatmap. (b) Clustering of SC metabolites identified in different tissues across three natural accessions. The metabolite content was transformed with min-max normalization and visualized by heatmap. (c) Principal component analysis (PCA) of SCs in four different tissues across three natural accessions. (d) Venn diagram showing the number of differential metabolites between compared groups. Samples of AR from three different geographical origins, including transverse sections from Jiangsu (JAR, J), Shaanxi (SAR, S), and Hubei (HAR, H). Secretory cavities in cortex (Co-SC), phloem (Ph-SC), xylem (Xy-SC), and pith (Pi-SC) are indicated. The colour scales in (a) and (b) indicate the relative change in metabolite levels (n=6).


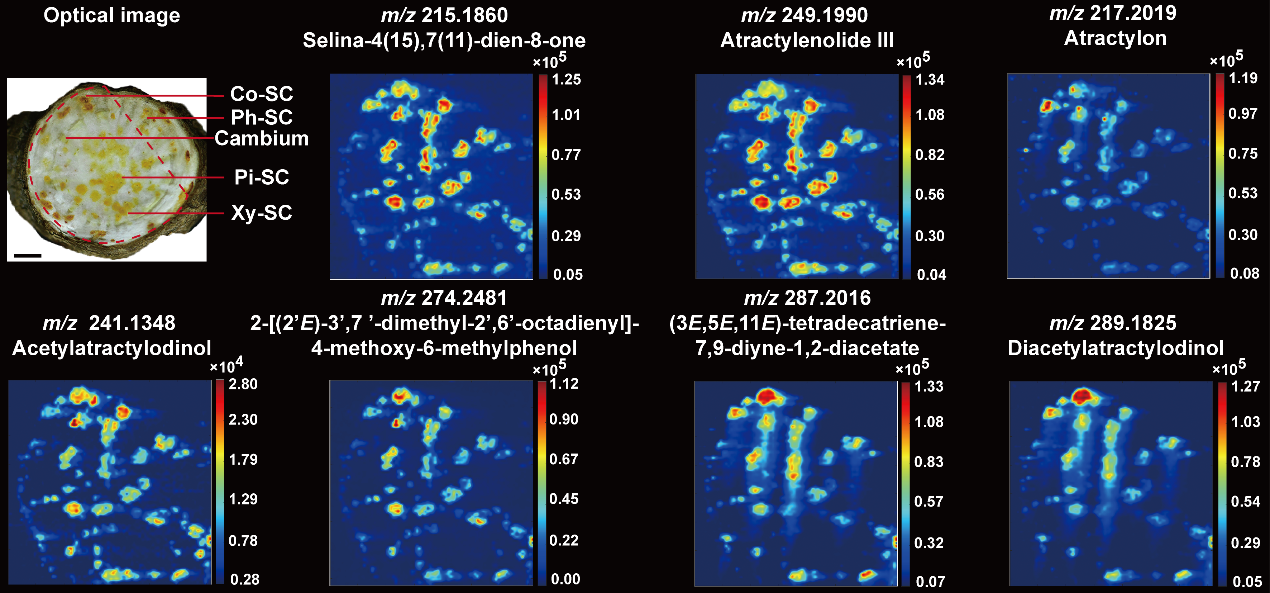


**Figure S7. DESI/PI-MSI of** **selected polyacetylenes and sesquiterpenes from rhizome section of Jiangsu *Atractylodes lancea* rhizome (JAR).** The optimal images of rhizome cross sections of JAR (selected trapezoid patch is marked with red dash line) and corresponding DESI/PI-MSI images of selected ions (m/z), including *m/z* 215.1860 (selina-4(15),7(11)-dien-8-one), *m/z* 249.1990 (atractylenolide III), *m/z* 217.2019 (atractylon), *m/z* 241.1348 (acetylatractylodinol), *m/z* 274.2481 (2-[(2’E)-3’,7’-dimethyl-2’,6’-octadienyl]-4-methoxy-6-methylphenol), *m/z* 287.2016 ((3E,5E,11E)-tetradecatriene-7,9-diyne-1,2-diacetate), *m/z* 289.1825 (diacetylatractylodinol). MS images were recorded with a scanning step size of 200 μm. Bar = 3 mm.


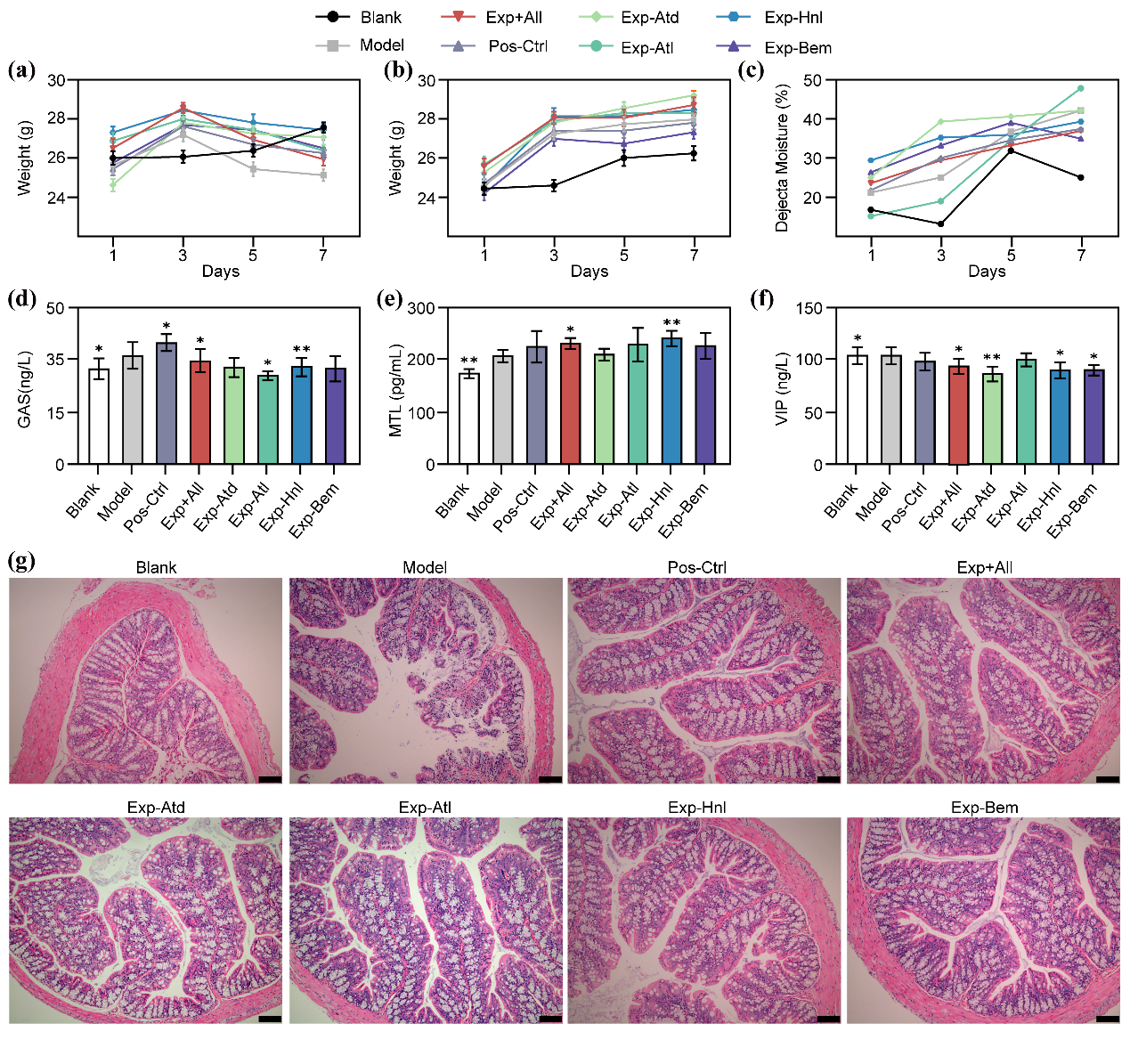


**Figure S8. The therapeutic effects of various proportions of essential oil components from *A. lancea* on digestive disorders in mice.** (a) Changes in body weight of mice during modelling (mean ± sem, n=8). (b) Changes in body weight of mice during the administration period (mean ± sem, n=8). (c) Changes in fecal moisture of mice during the administration period. (d-f) The effects of different ratios of essential oil components on the levels of Motilin (MTL), Gastrin (GAS), and Vasoactive Intestinal Peptide (VIP) in mouse serum (mean ± sem, n=8). Stars indicate significant differences to the model group (*t*-test, ^**^*P* < 0.01, ^*^*P* < 0.05). (g) Histopathological changes in mouse colonic tissue (Haematoxylin and Eosin staining, ×100). Bars = 100 μm. The experimental group Exp+All mirrors the compound composition found in Jiangsu Atractylodes lancea rhizome (JAR) from the geographic region of Geo-herb, featuring 44% Atractylon, 37% atractylodin, 8% hinesol, and 11% *ß*-eudesmol. In contrast, Exp-Atd excludes atractylodin, while Exp-Atl omits atractylon. Exp-Hnl eliminates hinesol from the mixture, and Exp-Bem removes *ß*-eudesmol.


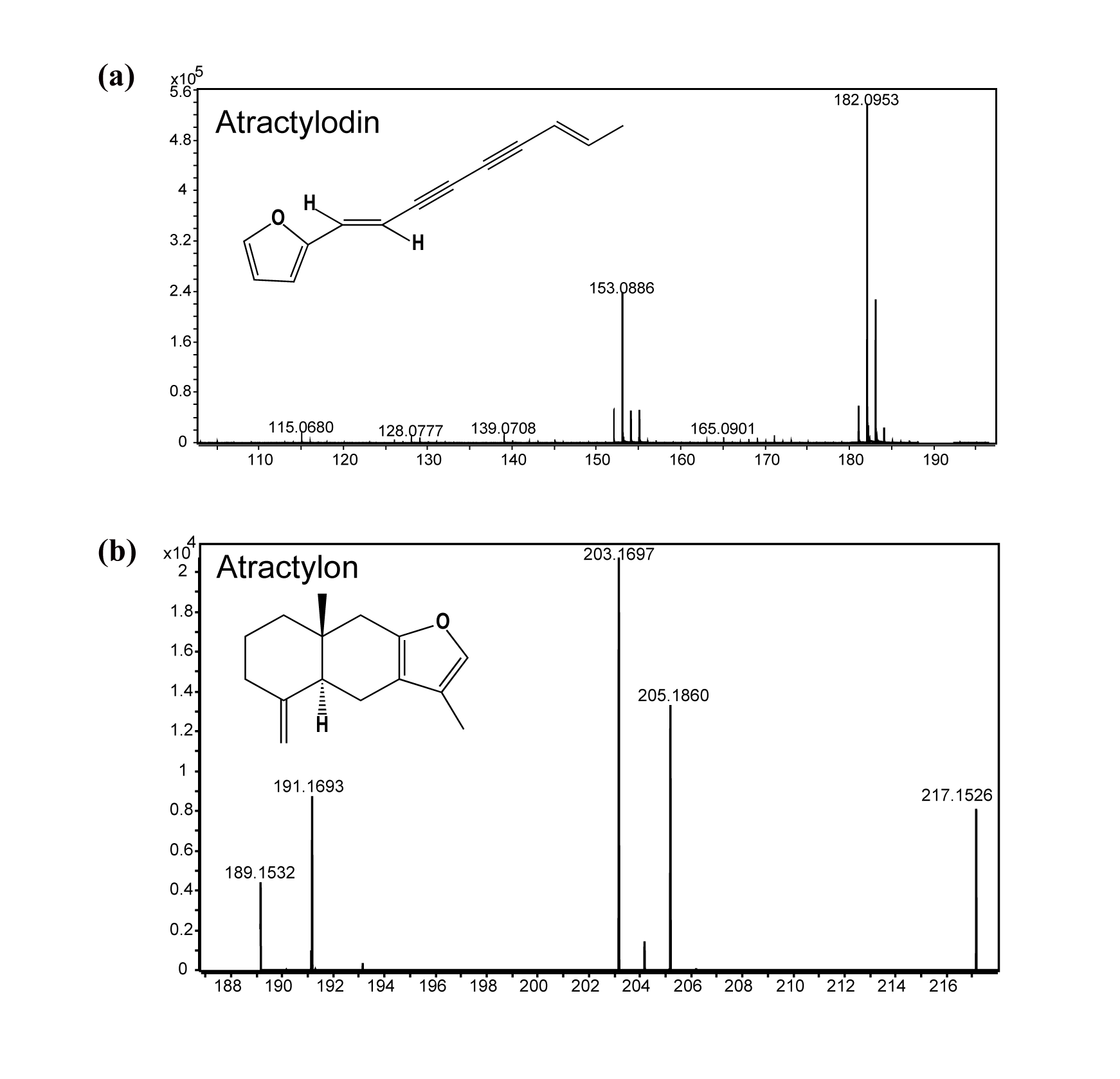


**Figure S9.** The DESI/PI mass spectra of the (a) atractylodin standard and (b) atractylon standard.
